# Operating Systems for Wireless Sensor Networks: A Survey

**DOI:** 10.3390/s110605900

**Published:** 2011-05-31

**Authors:** Muhammad Omer Farooq, Thomas Kunz

**Affiliations:** Department of Systems and Computer Engineering, Carleton University Ottawa, Canada; E-Mail:omer.farooq@ymail.com

**Keywords:** Wireless Sensor Network (WSN), Operating Systems (OS), embedded operating system, Real-Time Operating System (RTOS)

## Abstract

This paper presents a survey on the current state-of-the-art in Wireless Sensor Network (WSN) Operating Systems (OSs). In recent years, WSNs have received tremendous attention in the research community, with applications in battlefields, industrial process monitoring, home automation, and environmental monitoring, to name but a few. A WSN is a highly dynamic network because nodes die due to severe environmental conditions and battery power depletion. Furthermore, a WSN is composed of miniaturized motes equipped with scarce resources e.g., limited memory and computational abilities. WSNs invariably operate in an unattended mode and in many scenarios it is impossible to replace sensor motes after deployment, therefore a fundamental objective is to optimize the sensor motes’ life time. These characteristics of WSNs impose additional challenges on OS design for WSN, and consequently, OS design for WSN deviates from traditional OS design. The purpose of this survey is to highlight major concerns pertaining to OS design in WSNs and to point out strengths and weaknesses of contemporary OSs for WSNs, keeping in mind the requirements of emerging WSN applications. The state-of-the-art in operating systems for WSNs has been examined in terms of the OS Architecture, Programming Model, Scheduling, Memory Management and Protection, Communication Protocols, Resource Sharing, Support for Real-Time Applications, and additional features. These features are surveyed for both real-time and non-real-time WSN operating systems.

## 1. Introduction

**Figure 1.**
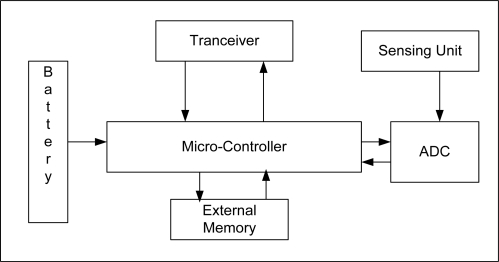
Sensor Node Architecture.

Advances in Micro-Electro Mechanical System (MEMS)-based sensor technology has led to the development of miniaturized and cheap sensor nodes, capable of communicating wirelessly, sensing and performing computations. A wireless sensor node is composed of a micro-controller, transceiver, timer, memory and analog to digital converter. Figure 1 shows the block diagram of a typical node. Sensor nodes are deployed to monitor a multitude of natural and man-made phenomena, *i.e*., habitant monitoring, wildlife monitoring, patient monitoring, industrial process monitoring and control, battle field surveillance, traffic control, and home automation, to name but a few. The main and most critical resources are energy (which is typically provided by a battery) and very limited main memory, with often allows storing only a few kilobytes. The micro-controller used in a wireless sensor node operates at low frequency compared to traditional contemporary processing units. These resource-constrained sensors are an impressive example of a System on Chip (SoC). Dense deployment of sensor nodes in the sensing field and distributed processing through multi-hop communication among sensor nodes is required to achieve high quality and fault tolerance in WSNs. Application areas for sensors are growing and new applications for sensor networks are emerging rapidly.

The OS acts as a resource manager for complex systems. In a typical system these resources include processors, memories, timers, disks, mice, keyboard, network interfaces, *etc*. The job of the OS is to manage the allocation of these resources to users in an orderly and controlled manner. Application programmers can then invoke different OS services through system calls. An OS multiplexes system resources in two ways *i.e*., in time and in space. Time multiplexing involves different programs taking turn in using the resources. Space multiplexing involves different programs accessing parts of the resource, possibly at the same time. Considering the resource constraints of typical sensor nodes in a WSN, a new approach is required for OS design in WSN.

Literature exists that surveys the application, transport, network and Medium Access Control (MAC) protocols for WSN and their application area, one such survey is [1]. A survey on Operating Systems for WSN also exists and is published in [2]. Since [2] was published, many new features have been introduced in contemporary operating systems for WSN, hence the need for this survey to update the previous one.

In this survey, we have examined the core OS features, such as its Architecture, Programming Model, Scheduling, Memory Management and Protection, Communication Protocols, Resource Sharing, and Support for Real-Time Applications, in both real-time and non-real-time WSN OSs. We also address different design approaches taken by different WSN OSs and discuss their relative pros and cons. The paper focuses on OS for severely resource-constraint WSN nodes such as motes, therefore we only survey OSs that have been designed for this class of devices. More powerful nodes, often acting as cluster heads and sinks in a WSN, can run more powerful and traditional OSs, for example the Sun Sunspot platform, or embedded Linux for iMotes and Stargate platforms. This paper concludes by setting forth future research directions for designing a WSN OS.

The remainder of this paper is organized as follows. In Section 2, we present major design concerns for a WSN OS. The importance of an OS to adequately support QoS in a WSN is discussed in Section 3. The next five sections review popular and new OSs for WSN: TinyOS is analyzed in Section 4, Section 5 describes the Contiki operating system, Section 6 presents the MANTIS Operating System, Nano-RK and LiteOS are surveyed in Sections 7 and 8 respectively. A comparative analysis follows in Section 9, future research directions are discussed in Section 10 and finally, Section 11 concludes this article.

## 2. Major Concerns in WSN OS Design

This section discusses details of major issues related to the design of a WSN OS.

### 2.1. Architecture

The organization of an OS constitutes its structure. The architecture of an OS has an influence on the size of the OS kernel as well as on the way it provides services to the application programs. Some of the well known OS architectures are the monolithic architecture, the micro-kernel architecture, the virtual machine architecture and the layered architecture.

A monolithic architecture in fact does not have any structure. Services provided by an OS are implemented separately and each service provides an interface for other services. Such an architecture allows bundling of all the required service together into a single system image, thus results in a smaller OS memory footprint. An advantage of the monolithic architecture is that the module interaction costs are low. Disadvantages associated with this architecture are: the system is hard to understand and modify, unreliable, and difficult to maintain. These disadvantages associated with monolithic kernels make them a poor OS design choice for contemporary sensor nodes.

An alternate choice is a microkernel architecture in which minimum functionality is provided inside the kernel. Thus, the kernel size is significantly reduced. Most of the OS functionality is provided via user-level servers like a file server, a memory server, a time server, *etc*. If one server fails, the whole system does not crash. The microkernel architecture provides better reliability, ease of extension and customization. The disadvantage associated with a microkernel is its poor performance because of frequent user to kernel boundary crossings. A microkernel is the design choice for many embedded OS due to the small kernel size and the number of context switches in a typical WSN application is considered to be far fewer. Thus, fewer boundary crossing are required compared to traditional systems.

A virtual machine is another architectural choice. The main idea is to export virtual machines to user programs, which resemble hardware. A virtual machine has all the needed hardware features. The key advantage is its portability and a main disadvantage is typically a poor system performance.

A layered OS architecture implements services in the form of layers. Advantages associated with the layered architecture are: manageability, easy to understand, and reliability. A main disadvantage is that it is not a very flexible architecture from an OS design perspective.

An OS for a Wireless Sensor Network should have an architecture that results in a small kernel size, hence small memory footprint. The architecture must allow extensions to the kernel if required. The architecture must be flexible *i.e*., only application-required services get loaded onto the system.

### 2.2. Programming Model

The programming model supported by an OS has a significant impact on the application development. There are two popular programming models provided by typical WSN OSs, namely: event driven programming and multithreaded programming. Multithreading is the application development model most familiar to programmer, but in its true sense rather resource intensive, therefore not considered well suited for resource constraint devices such as sensor nodes. Event driven programming is considered more useful for computing devices equipped with scarce resource but not considered convenient for traditional application developers. Therefore researchers have focused their attention on developing a light-weight multithreading programming model for WSN OSs. Many contemporary WSN OSs now provide support for the multithreading programming model and we discuss them in detail later.

### 2.3. Scheduling

The Central Processing Unit (CPU) scheduling determines the order in which tasks are executed on a CPU. In traditional computer systems, the goal of a scheduler is to minimize latency, to maximize throughput and resource utilization, and to ensure fairness.

The selection of an appropriate scheduling algorithm for WSNs typically depends on the nature of the application. For applications having real-time requirements, real-time scheduling algorithm must be used. For other applications, non-real-time scheduling algorithms are sufficient.

WSNs are being used in both real-time and non-real-time environments, therefore a WSN OS must provide scheduling algorithms that can accommodate the application requirements. Moreover, a suitable scheduling algorithm should be memory and energy efficient.

### 2.4. Memory Management and Protection

In a traditional operating system, memory management refers to the strategy used to allocate and de-allocate memory for different processes and threads. Two commonly used memory management techniques are static memory management and dynamic memory management. Static memory management is simple and it is a useful technique when dealing with scare memory resources. At the same time, it results in inflexible systems because run-time memory allocation cannot occur. On the other hand, dynamic memory management yields a more flexible system because memory can be allocated and de-allocated at run-time. Process memory protection refers to the protection of one process’ address space from another. In early sensor network operating systems like TinyOS [3] there was no memory management available. Initial operating systems for WSNs assumed that only a single application executes on a sensor mote, therefore there is no need for memory protection. With the emergence of new application domains for WSNs, contemporary WSNs provides support for multiple threads of execution, consequently memory management becomes an issue for WSN OS.

### 2.5. Communication Protocol Support

In the OS context, communication refers to inter-process communication within the system as well as with other nodes in the network. WSNs operate in a distributed environment, where senor nodes communicate with other nodes in the network. All WSN OSs provide an Application Programming Interface (API) that enables application program to communicate. It is possible that a WSN is composed of heterogeneous sensor nodes, therefore the communication protocol provided by the OS must also consider heterogeneity. In network-based communication, the OS should provide transport, network, and MAC layer protocol implementations.

### 2.6. Resource Sharing

The responsibility of an OS includes resources allocation and resource sharing, which is of immense importance when multiple programs are concurrently executing. The majority of WSNs OSs today provide some sort of multithreading, requiring a resource sharing mechanism. This can be performed in time e.g., scheduling of a process/thread on the CPU and in space e.g., writing data to system memory. In some cases, we need serialized access to resources and this is done through the use of synchronization primitives.

## 3. Support for Real-Time Applications

A WSN can be used to monitor a mission critical system. Therefore an OS for WSN should provide implementations of real-time scheduling algorithms to meet the deadlines of hard real-time tasks. With the advent of Wireless Multimedia Sensor Networks (WMSNs) [4], an OS for WSN should provide implementations of communication protocols that support real-time multimedia streams. For example, an OS can provide an implementation of a MAC protocol that reduces the end to end delay of multimedia streams, moreover OS designers should strive to provide implementations of real-time communication protocols at the network and transport layers. Furthermore, an OS for WSN should provide an Application Programming Interface (API) to the application programmer that allows them to implement custom communication protocols on top of the communication protocol stack supported by the OS. Above all the OS must provide an implementation of a QoS Architecture for traffic segregation at the network layer.

The decreasing cost of hardware such as CMOS cameras and microphones has resulted in a new variant of WSNs called Wireless Multimedia Sensor Nodes or WMSNs. WMSN nodes are equipped with integrated cameras, microphones, and scalar sensors. Such sensor nodes are capable of capturing and communicating audio and video streams over a wireless channel. WSMNs are more sophisticated as compared to ordinary sensor nodes, but still have limited resources, compared to contemporary computing platforms. Primarily, WSNs and WMSNs are intended to capture and transmit information ubiquitously to the sink nodes present in the network. Hence, the communication protocol plays a pivotal role for correct functionality of such networks. Scarce resources and the wireless communication medium inhibit the use of the traditional layered architecture like the TCP/IP protocol stack in WSNs [5]. Secondly, TCP was designed for wired networks and its performance in wireless communication is reported to be poor. Moreover, in case of WMSNs, TCP is not a recommended protocol for multimedia applications mainly due to its flow and congestion control mechanisms. UDP can be an alternative for TCP for multimedia applications but it does not provide any feedback about the status of the network that may be required for proper transmission of multimedia data. Hence, both TCP and UDP do not serve as ideal transport layer protocols for WMSNs.

The cross layer architecture design paradigm is emerging as a promising technique for networking using wireless communication. In cross layer design, depending upon the condition of a wireless link, the MAC layer can choose appropriate error coding techniques. Similarly, the network layer can choose a path by taking input from the application and the physical layer. A cross layer architecture can adapt the behavior of the protocol stack to the requirements of the application; or, in the reverse direction, it can adapt the behavior of an application to the physical link conditions. So far, it has been assumed that WSNs run only a single application on a sensor node. As a result, researchers have developed cross layer architectures that can work efficiently for single application wireless sensor networks.

In the light of the above discussion it can be seen that new emerging application areas of WSNs place additional demands on the OS. Due to the resource limitations and the nature of the wireless link, the cross layer protocol design approach has been reported to work well. Therefore, a contemporary OS for WSN should provide an implementation of the protocol stack that enables cross layer interaction and fine-tunes the parameters of the protocol stack in accordance with the application requirements. An OS must support a transport layer protocol that supports real-time applications. The real-time transport protocol should monitor the network conditions and minimize congestion inside the network so that real-time flows experience acceptable quality. Furthermore, the OS needs to provide an implementation of a routing protocol that constructs route according to the QoS requirements of applications. Moreover, it must support a MAC algorithm that schedules packets w.r.t. their priority.

## 4. TinyOS

TinyOS [3] is an open source, flexible, component based, and application-specific operating system designed for sensor networks. TinyOS can support concurrent programs with very low memory requirements. The OS has a footprint that fits in 400 bytes. The TinyOS component library includes network protocols, distributed services, sensor drivers, and data acquisition tools. The following subsections survey the TinyOS design in more detail.

### 4.1. Architecture

**Figure 2.**
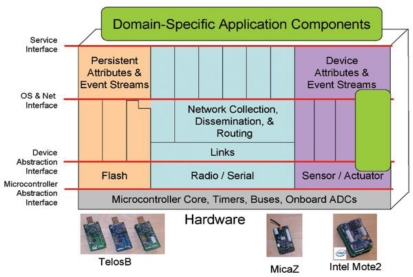
TinyOS Architecture.

TinyOS falls under the monolithic architecture class. TinyOS uses the component model and, according to the requirements of an application, different components are glued together with the scheduler to compose a static image that runs on the mote. A component is an independent computational entity that exposes one or more interfaces. Components have three computational abstractions: commands, events, and tasks. Mechanisms for inter-component communication are commands and events. Tasks are used to express intra-component concurrency. A command is a request to perform some service, while the event signals the completion of the service. TinyOS provides a single shared stack and there is no separation between kernel space and user space. Figure 2 shows the TinyOS architecture.

### 4.2. Programming Model

Earlier versions of TinyOS did not provide any multithreading support, with application development strictly following the event driven programming model. TinyOS version 2.1 provides support for multithreading and these TinyOS threads are called TOS Threads. In [6], the authors pointed out the problem that, given the motes’ resource constraints, an event-based OS permits greater concurrency. However, preemptive threads offer an intuitive programming paradigm. The TOS threading package provides the ease of a threading programming model coupled with the efficiency of an event driven kernel. TOS threads are backward compatible with existing TinyOS code. TOS threads use a cooperative threading approach, *i.e*., TOS threads rely on applications to explicitly yield the processor. This adds an additional burden on the programmer to explicitly manage the concurrency. Application level threads in TinyOS can preempt other application level threads but they cannot preempt tasks and interrupt handlers. A high priority kernel thread is dedicated to running the TinyOS scheduler. For communication between the application threads and the kernel, TinyOS 2.1 provides message passing. When an application program makes a system call, it does not directly execute the code. Rather it posts a message to the kernel thread by posting a task. Afterwards, the kernel thread preempts the running thread and executes the system call. This mechanism ensures that only the kernel directly executes TinyOS code. System calls like *Create*, *Destroy*, *Pause*, *Resume and Join* are provided by the TOS threading library.

TOS threads dynamically allocate Thread Control Blocks (TCB) with space for a fixed size stack that does not grow over time. TOS Threads context switches and system calls introduce an overhead of less than 0.92% [3].

Earlier versions of TinyOS imposed atomicity by disabling the interrupts, *i.e*., telling the hardware to delay handing the external events until after the application completed an atomic operation. This scheme works well on uniprocessor systems. Critical section can occur in the user level threads and the designer of the OS does not want the user to disable the interrupts due to system performance and usability issues. This problem is circumvented in TinyOS version 2.1. It provides synchronization support with the help of condition variables and mutexes. These synchronization primitives are implemented with the help of special hardware instructions e.g., test & set instruction.

### 4.3. Scheduling

Earlier versions of TinyOS supported a non-preemptive First-In-First-Out (FIFO) scheduling algorithm. Therefore, those versions of TinyOS do not support real-time application. The core of the TinyOS execution model are tasks that run to completion in a FIFO manner. Since TinyOS supports only non preemptive scheduling, task must obey run to completion semantics. Tasks run to completion with respect to other task but they are not atomic with respect to interrupt handlers, commands, and events they invoke. Since TinyOS uses FIFO scheduling, disadvantages associated with FIFO scheduling are also associated with the TinyOS scheduler. The wait time for a task depends on the task’s arrival time. FIFO scheduling can be unfair to latter tasks especially when short tasks are waiting behind longer ones.

In [3], the authors claim that they have added support for an Earliest Deadline First (EDF) scheduling algorithm in TinyOS, to facilitate real-time applications. The EDF scheduling algorithm does not produce a feasible schedule when tasks content for resources. Thus, TinyOS does not provide a solid real-time scheduling algorithm if different threads content for resources.

### 4.4. Memory Management and Protection

In [7], efficient memory safety for TinyOS is presented. In sensor nodes, hardware-based memory protection is not available and the resources are scarce. Resource constraints necessitate the use of unsafe, low level languages like nesC [8]. In TinyOS version 2.1, memory safety is incorporated. The goals for memory safety as given in [7] are: trap all pointer and array errors, provide useful diagnostics, and provide recovery strategies. Implementations of memory-safe TinyOS exploits the concept of a Deputy. The Deputy is a resource to resource compiler that ensures type and memory safety for C code. Code compiled by Deputy relies on a mix of compile and run-time checks to ensure memory safety. Safe TinyOS is backward compatible with earlier version of TinyOS. The Safe TinyOS tool chain inserts checks into the application code to ensure safety at run-time. When a check detects that safety is about to be violated, code inserted by Safe TinyOS takes remedial actions. TinyOS uses a static memory management approach.

### 4.5. Communication Protocol Support

Earlier versions of TinyOS provide two multi-hop protocols: dissemination and TYMO [9,10]. The dissemination protocol reliably delivers data to every node in the network. This protocol enables administrators to reconfigure queries and to reprogram a network. The dissemination protocol provides two interfaces: *DisseminationValue* and *DisseminationUpdate*. A producer calls *DisseminationUpdate*. The command *DisseminationUpdate.change*() should be called each time the producers wants to disseminate a new value. On the other hand, the *DisseminationValue* interface is provided for the consumer. The event *DisseminationValue.changed*() is signaled each time the dissemination value is changed. TYMO is the implementation of the DYMO protocol, a routing protocol for mobile *ad hoc* networks. In TYMO, packet formats have changed and it has been implemented on top of the active messaging stack.

Lin *et al.* [11] have presented DIP, a new dissemination protocol for sensor networks. DIP is a data discovery and dissemination protocol that scales to hundreds of values. TinyOS version 2.1.1 now also provides support for 6lowpan [12], an IPv6 networking layer within a TinyOS network.

At the MAC layer, TinyOS provides an implementation of the following protocols: a single hop TDMA protocol, a TDMA/CSMA hybrid protocol which implements Z-MAC’s slot stealing optimization, B-MAC, and an optional implementation of an IEEE 802.15.4 complaint MAC.

### 4.6. Resource Sharing

TinyOS uses two mechanisms for managing shared resources: Virtualization and Completion Events. A virtualized resource appears as an independent instance. *i.e*., the application uses it independent of other applications. Resources that cannot be virtualized are handled through completion events. The GenericComm communication stack of TinyOS is shared among different threads and it cannot be virtualized. GenericComm can only send one packet at a time, send operations of other threads fail during this time. Such shared resources are handled through completion events that inform waiting threads about the completion of a particular task.

### 4.7. Support for Real-Time Applications

TinyOS does not provide any explicit support for real-time applications. As we already discussed in the scheduling section above, tasks in TinyOS observe run to completion semantics in a FIFO manner, hence in its original form, TinyOS is not a good choice for sensor networks that are being deployed to monitor real-time phenomena. An effort has been made to implement an Earliest Deadline First (EDF) process scheduling algorithm and it has been made available in newer versions of TinyOS. However, it has been shown that the EDF algorithm cannot produce a feasible schedule when tasks content for resources. In the nutshell, TinyOS is not a strong choice for real-time applications.

TinyOS does not provide any specific MAC, network, or transport layers protocol implementations that support Quality of Service requirements of real-time multimedia streams. At the MAC layer, TinyOS supports TDMA, which can be fine-tuned depending upon the requirements of an application to support multimedia traffic streams.

### 4.8. Additional Features

In this section, we discuss some additional features provided by TinyOS.

- **File System**
  TinyOS provides a single level file system. The rationale behind providing a single level file system is the assumption that only a single application runs on the node at any given point in time. As node memory is scarce, having a single level file system is therefore sufficient.
- **Database Support**
  The purpose of sensor nodes is to sense, perform computations, store and transmit data, therefore TinyOS provides database support in the form of TinyDB. Further details on TinyDB can be found in [13].
- **Security Support**
  Communication security in wireless broadcast medium is always required. TinyOS provides its communication security solution in the form of TinySec [14].
- **Simulation Support**
  TinyOS provides simulation support in the form of TOSSIM [15]. The simulation code is written in NesC and consequently can also be deployed to actual motes.
- **Language Support**
  TinyOS supports application development in the NesC programming language. NesC is a dialect of the C language.
- **Supported Platforms**
  TinyOS supports the following sensing platforms: Mica [16], Mica2 [16], Micaz [16], Telos [17], Tmote [17] and a few others.
- **Documentation Support**
  TinyOS is a well documented OS and extensive documentation can be found on the TinyOS home page at http://www.tinyos.net.

## 5. Contiki

Contiki [18], is a lightweight open source OS written in C for WSN sensor nodes. Contiki is a highly portable OS and it is build around an event-driven kernel. Contiki provides preemptive multitasking that can be used at the individual process level. A typical Contiki configuration consumes 2 kilobytes of RAM and 40 kilobytes of ROM. A full Contiki installation includes features like: multitasking kernel, preemptive multithreading, proto-threads, TCP/IP networking, IPv6, a Graphical User Interface, a web browser, a personal web server, a simple telnet client, a screensaver, and virtual network computing.

### 5.1. Architecture

The Contiki OS follows the modular architecture. At the kernel level it follows the event driven model, but it provides optional threading facilities to individual processes. The Contiki kernel comprises of a lightweight event scheduler that dispatches events to running processes. Process execution is triggered by events dispatched by the kernel to the processes or by a polling mechanism. The polling mechanism is used to avoid race conditions. Any scheduled event will run to completion, however, event handlers can use internal mechanisms for preemption.

Two kinds of events EW supported by Contiki OS: asynchronous events and synchronous events. The difference between the two is that synchronous events are dispatched immediately to the target process that causes it to be scheduled. On the other hand asynchronous events are more like deferred procedure calls that are en-queued and dispatched later to the target process.

The polling mechanism used in Contiki can be seen as high-priority events that are scheduled in between each asynchronous event. When a poll is scheduled, all processes that implement a poll handler are called in order of their priority.

**Figure 3.**
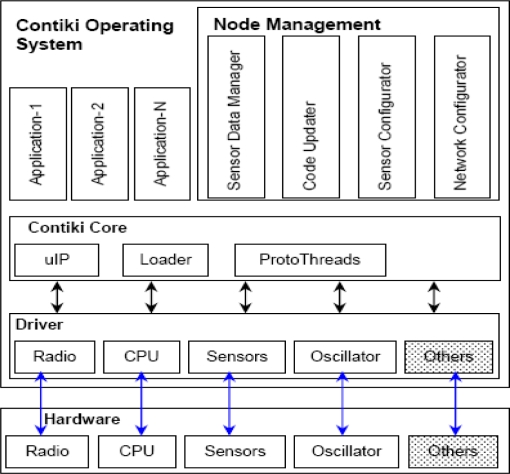
Contiki Architecture.

All OS facilities e.g., senor data handling, communication, and device drivers are provided in the form of services. Each service has its interface and implementation. Applications using a particular service need to know the service interface. An application is not concerned about the implementation of a service. Figure 3 shows the block diagram of the Contiki OS architecture, as given in [19].

### 5.2. Programming Model

Contiki supports preemptive multithreading. Multi-threading is implemented as a library on top of the event-driven kernel. The library can be linked with applications that require multithreading. The Contiki multithreading library is divided in two parts: a platform independent part and a platform specific part. The platform independent part interfaces to the event kernel and the platform specific part of the library implements stack switching and preemption primitives. Since preemption is supported, preemption is implemented using the timer interrupt and the thread state is stored on a stack.

For multithreading, Contiki uses protothreads [20]. Protothreads are designed for severely memory constraint devices because they are stack-less and lightweight. The main features of protothreads are: very small memory overhead (only two bytes per protothread), no extra stack for a thread, highly portable (*i.e*., they are fully written in C and hence there is no architecture-specific assembly code). Since events run to completion and Contiki does not allow interrupt handlers to post new events, no process synchronization is provided in Contiki.

### 5.3. Scheduling

Contiki is an event-driven OS, therefore it does not employ any sophisticated scheduling algorithm. Events are fired to the target application as they arrive. In case of interrupts, interrupt handlers of an application runs w.r.t. their priority.

### 5.4. Memory Management and Protection

Contiki supports dynamic memory management. Apart from this it also supports dynamic linking of the programs. In order to guard against memory fragmentation Contiki uses a Managed Memory Allocator [21]. The primary responsibility of the managed memory allocator is to keep the allocated memory free from fragmentation by compacting the memory when blocks are free. Therefore, a program using the memory allocator module cannot be sure that allocated memory stays in place.

For dynamic memory management, Contiki also provides memory block management functions [21]. This library provides simple but powerful memory management functions for blocks of fixed length. A memory block is statically declared using the MEMB() macro. Memory blocks are allocated from the declared memory by the memb_alloc() function, and are de-allocated using the memb_free() function.

It is worth noting here that Contiki does not provide any memory protection mechanism between different applications.

### 5.5. Communication Protocol Support

Contiki supports a rich set of communication protocols. In Contiki, an application can use both versions of IP *i.e*., IPv4 and IPv6. Contiki provides an implementation of *u*IP, a TCP/IP protocol stack for small 8 bit micro-controllers. *u*IP does not require its peers to have a complete protocol stack, but it can communicate with peers running a similar lightweight stack. The *u*IP implementation has the minimum set of features needed for a full TCP/IP stack. *u*IP is written in C, it can only support one network interface, and it supports TCP, UDP, ICMP, and IP protocols.

Contiki provides another lightweight layered protocol stack, called Rime, for network-based communication. Rime provides single hop unicast, single hop broadcast, and multi-hop communication support. Rime supports both best effort and reliable transmission. In multi-hop communication, Rime allows applications to run their own routing protocols. Applications are allowed to implement protocols that are not present in the Rime stack.

Contiki does not support multicast. Therefore Contiki does not provide any implementation of group management protocols such as the Internet Group Management Protocol (ICMP), or Multicast Listener Discovery (MLD) protocol.

Since memory is a scare resource in embedded devises, *u*IP uses memory efficiently by using memory management mechanisms. The *u*IP stack does not use explicit dynamic memory allocation. It uses a global buffer to hold the incoming data packets. Whenever a packet is received, Contiki places it in the global buffer and notifies the TCP/IP stack. If it is a data packet, TCP/IP notifies the appropriate application. The application needs to copy the data in the secondary buffer or it can immediately process the data. Once the application is done with the received data, Contiki overwrites the global buffer with new incoming data. If an application delays data processing, then data can be overwritten by new incoming data packets.

Contiki provides an implementation of RPL (IPv6 routing protocol for low power and lossy networks) [22] by the name ContikiRPL [23]. ContikiRPL operates over low power wireless links and lossy power line links.

### 5.6. Resource Sharing

Since events run to completion and Contiki does not allow interrupt handlers to post new events, Contiki provides serialized access to all resources.

### 5.7. Support for Real-Time Applications

Contiki does not provide any support for real-time applications, hence there is no implementation of any real-time process scheduling algorithm in Contiki. On the network protocol stack side, Contiki does not provide any protocol that considers the QoS requirements of multimedia applications. Furthermore, since Contiki provides an implementation of the micro IP stack, interactions between different layers of the protocol stack are not possible.

### 5.8. Additional Features

In this section, we briefly discuss additional features provided by the Contiki OS.

- **Coffee File System**
  Contiki provides file system support for flash-based sensor devices in the form of the Coffee file system [24]. The purpose of the Coffee file system is to provide a programming interface for building efficient and portable storage abstractions. Coffee provides a platform independent storage abstraction through an expressive programming interface. Coffee uses a small and constant RAM footprint per file, making it scalable. In default setup, Coffee requires 5 Kb ROM for the code and 0.5 Kb RAM at run-time. A simple sequential page structure is being used, Coffee also introduces the concept of micro logs to handle file modifications without using a spanning log structure. Because of the contiguous page structure file metadata, Coffee uses a small and constant footprint for each file. Flash memory is divided into logical pages and the size of the page typically matches the underlying flash memory pages. If the file size is not known beforehand, Coffee allocates a predefined amount of pages to the file. Later on, if the reserved size turns out to be insufficient, Coffee creates a new larger file and copies the old file data into it. To boost the file system performance, by default Coffee uses a metadata cache of 8 entries in the RAM. Coffee also provides an implementation of a garbage collector that reclaims obsolete pages when a file reservation request cannot be satisfied. To allocate pages to a file, Coffee uses a first fit algorithm. Flash memories suffer from wear, *i.e*., every time a page is erased it increases the chances of memory corruption. Coffee uses wear leveling and its purpose is to spread sector erasures evenly to minimize the risk of damaging some sectors much earlier than others. Coffee provides the following APIs to the application programmers. Open(), read(), modify(), seek(), append(),close(). Detailed description of these APIs can be found in the Contiki documentation.
- **Security Support**
  Contiki does not provide support for secure communication. A proposal and implementation of a secure communication protocol with the name ContikiSec has been provided in [25].
- **Simulation Support**
  Contiki provides sensor network simulations through Cooja [26].
- **Language Support**
  Cotiki supports application development in the C language.
- **Supported Platforms**
  Contiki supports the following sensing platforms: Tmote [17], AVR series MCU [27].
- **Documentation Support**
  Contiki documentation can be found on the Contiki home page at: http://www.sics.se/contiki.

## 6. MANTIS

The MultimodAl system for NeTworks of In-situ wireless Sensors (MANTIS) provides a new multithreaded operating system for WSNs. MANTIS is a lightweight and energy efficient operating system. It has a footprint of 500 bytes, which includes kernel, scheduler, and network stack. The MANTIS Operating System (MOS) key feature is that it is portable across multiple platforms, *i.e*., we can test MOS applications on a PDA or a PC [28]. Afterwards, the application can be ported to the sensor node. MOS also supports remote management of sensor nodes through dynamic programming. MOS is written in C and it supports application development in C. The following subsections discuss the design features of MOS in more detail.

### 6.1. Architecture

**Figure 4.**
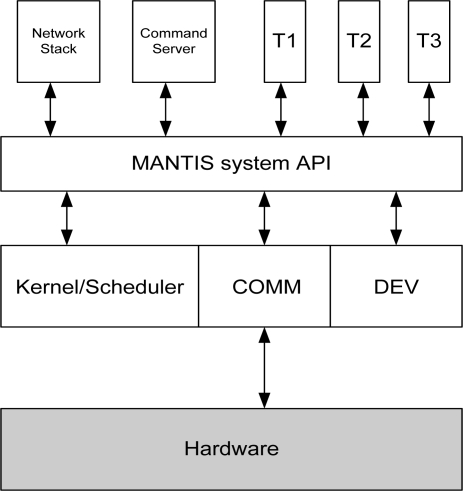
MANTIS OS Architecture. Kernel Scheduler, COMM, DEV, MANTIS System API, Network Stack, and Command Server comprises MOS.

MOS follows the layered architectural design as shown in Figure 4. In a layered architecture, services provided by an OS are implemented in layers. Each layer acts as an enhanced virtual machine to the layers above. Following are the different services implemented at each layer of MOS.

**Layer 3:** Network Stack, Command Server, and User Level Threads
**Layer 2:** MANTIS system API
**Layer 1:** Kernel/Scheduler, Communication Layer (MAC and PHY), and Device Drivers
**Layer 0:** Hardware

The MOS kernel only handles the timer interrupt, and all other interrupts are directly sent to associated device drivers. When a device driver receives an interrupt, it posts a semaphore in order to activate a waiting thread, and this thread handles the event that caused the interrupt.

### 6.2. Programming Model

MOS supports preemptive multitasking. The MOS team designed a multithreaded OS because of the facts presented in [29], i.e., “*A thread driven system can achieve the high performance of event based systems for concurrency intensive applications, with appropriate modification to the threading package*.” Sensor node memory is a scare resource, therefore MOS maintains two logically distinct sections of RAM: the space for global variables that is allocated at compile time, while the rest of the RAM is managed as a heap. Whenever a thread is created, stack space is allocated by the kernel from the heap. The stack space is returned to the heap once the thread exits. The thread table is the main data structure that is being managed by the MOS kernel. In the thread table, there is a one entry per thread. MOS statically allocates memory for the thread table, therefore there can only be fixed maximum number of threads, on the other hand the overhead of the thread table is fixed. The maximum number of threads can be adjusted at the compile time, by default it is 12. The thread table entry comprises 10 bytes and it contains: current stack pointer, stack boundary information (base pointer and size), pointer to thread starting function, thread priority level, and pointer to next thread. Once a thread is suspended, its context is saved on the stack. Since each thread table entry comprises 10 bytes and by default 12 threads can be created, the associated overhead in terms of memory is 120 bytes. By default each thread gets a time slice of 10 ms and a context switch happens with the help of timer interrupts. System calls and posting of a semaphore operation can also trigger a context switch.

Multithreading support in MOS comes at the cost of context switching and stack memory overhead. In [28], the argument presented in favor of context switching overhead is that it is only a moderate issue in WSNs. It has been observed that each context switch incurs 60 microseconds overhead. In comparison to this, the default time slice is much larger *i.e*., 10 ms, so the context switch overhead is less than 1%. A second cost is the stack memory allocation. The default thread stack in MOS is 128 bytes and MICA2 motes have a 4 KB RAM. Since the MOS kernel occupies only 500 bytes, considerable space is available to support multithreading.

MOS avoids race conditions by using binary mutexes and counting semaphores. A semaphore in MOS is a 5 byte structure and it is declared by an application as needed. The semaphore structure contains a lock or count byte along with head and tail pointers.

### 6.3. Scheduling

MOS uses preemptive priority-based scheduling. MOS uses a UNIX-like scheduler with multiple priority classes and it uses the round robin approach within each priority class. The length of time slice is configurable, by default it is set to 10 milliseconds (ms). The scheduler uses a timer interrupt for context switches. Context switches are also triggered by system calls and semaphore operations. Energy efficiency is achieved by the MOS scheduler by switching the microcontroller to sleep mode when application threads are idle.

The ready queue of the MOS scheduler has five priorities, ranging from high to low: Kernel, Sleep, High, Normal, and Idle. The scheduler schedules the highest priority task in the ready queue. The task either runs to completion or gets preempted if its time slice expires. For time slicing, the MOS scheduler uses a 16 bit timer. When there is no thread in the ready queue, the system goes to sleep mode. If the system is suspended on I/O, the system enters the moderate idle sleep mode. If the application threads have called the sleep() system call, the system switches into a deep power save sleep mode. A separate queue maintains the ordered list of threads that have called sleep(), and is ordered by sleep time from low to high. The sleep priority in the ready queue enables newly awoken threads to have the highest priority, so that they can be serviced first after they wake up.

The MOS kernel maintains ready list head and tail pointers for each priority level. There are five priority levels and these pointers consume 20 bytes in total. These two pointers helps in fast addition and deletion of threads from a ready queue, hence improve performance in manipulating threads list. MOS also uses a current thread pointer of 2 bytes, an interrupt status byte, and one byte of flags. The total static overhead for scheduling is 144 bytes.

The MOS scheduler uses round robin scheduling within the each priority class. This means threads of the highest priority class can cause lower priority class threads to starve. MOS use priority scheduling that may support real-time task better than the TinyOS or Contiki schedulers. But it still requires real-time schedulers like Rate Monotonic and Earliest Deadline First in order to truly accommodate real-time tasks.

### 6.4. Memory Protection and Management

MANTIS allows dynamic memory management but it discourages its use because dynamic memory management incurs lots of overhead. Secondly, memory is a scarce resource in a sensor node. MANTIS manages different threads’ memory using the thread table that has already been discussed. MANTIS does not provide any mechanism for memory protection.

### 6.5. Communication Protocol Support

MOS implements the network stack in two parts. The first part of the network protocol stack is implemented in user space, as shown in Figure 4. That part contains the implementation of layer 3 (and above) protocols. A second part contains the implementation of the MAC and PHY layer operations. The rationale behind implementing the layer 3 and above functionality in user space is to provide flexibility. If an application wants to use its own data-driven routing protocol, then it can implement its routing protocol in user space. The downside of the approach is performance *i.e*., the network protocol stack has to use APIs provided by MANTIS instead of communicating directly with the device driver and hardware. This results in many context switches, resulting in computational and memory overheads.

The second part of the networking protocol stack is implemented in a COMM layer. The COMM layer primarily implements synchronization and MAC layer functionalities. The COMM layer provides a unified interface for communication with device drivers, for interfaces such as serial connections, USB, and radio devices. The COMM layer also performs packet buffering. It is possible that packets arrive from the network for a thread that is not currently scheduled. In such scenarios the COMM layer will buffer packets. Once the thread gets scheduled, the COMM layer passes a pointer to the data to the concerned thread.

MANTIS OS does not provide support for multicast applications, furthermore it does not provide an implementation for group management protocols. MANTIS also does not provide support for real-time multimedia applications in its communication protocol stack. On the other hand MANTIS does provide a facility to implement custom routing and transport layer protocols on top of the MAC layer. Hence, one can implement real-time transport and routing protocols for multimedia sensor networks in MANTIS.

### 6.6. Resource Sharing

MANTIS performs resource sharing with the help of semaphores. At the same time it does not address the priority inversion phenomenon, where a higher priority process waits on a lower priority process.

### 6.7. Support for Real-Time Applications

MANTIS provides very little support for real-time applications at the process level. It has been discussed above that MOS uses priority scheduling within each priority class. Thus processes running high priority tasks can be mapped to the higher priority class and within that process class such processes can further be assigned high priorities. MOS does not provide an implementation of a scheduling algorithm that can meet soft and hard deadlines of processes. Therefore, MOS is not a real-time OS for WSNs. For real-time multimedia applications, additional functionalities are required in the network protocol stack e.g., allocating network bandwidth to multimedia applications, finding routes that can satisfy QoS requirements of flows, *etc*. MOS does not provide any such functionality in its network protocol stack.

### 6.8. Additional Features

In this section we discuss the additional features provided by the MANTIS OS.

- **Simulation Support**
  MANTIS supports wireless sensor network simulation through AVRORA [30].
- **Language Support**
  MANTIS supports application development in the C language.
- **Supported Platforms**
  MANTIS supports the following sensing platforms: Mica2 [16], MicaZ [16], and Telos [17].
- **Shell**
  An implementation of a Unix-like shell comes with MANTIS that runs on the sensor node.
- **Documentation Support**
  MANTIS documentation can be found on the MANTIS home page at: http://mantisos.org

## 7. Nano-RK

Nano-RK [31] is a fixed, preemptive multitasking real-time OS for WSNs. The design goals for Nano-RK are multitasking, support for multi-hop networking, support for priority-based scheduling, timeliness and schedulability, extended WSN lifetime, application resource usage limits, and small footprint. Nano-RK uses 2 Kb of RAM and 18 Kb of ROM. Nano-RK provides support for CPU, sensors, and network bandwidth reservations. Nano-RK supports hard and soft real-time applications by the means of different real-time scheduling algorithms, e.g., rate monotonic scheduling and rate harmonized scheduling [32]. Nano-RK provides networking support through socket-like abstraction. Nano-RK supports FireFly [33] and MicaZ sensing platforms.

### 7.1. Architecture

**Figure 5.**
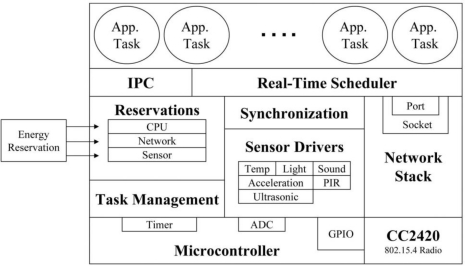
Nano-RK Architecture.

Nano-RK follows the monolithic kernel architecture model. Due to its real-time nature, the authors of Nano-RK emphasis the use of a static design time framework *i.e*., task priorities, deadlines, period, and their reservations should be assigned offline, so that admission control procedures can be applied efficiently. By choosing this static approach, one can determine whether the task deadlines can be met in the overall system design or not. Application programmers can change different parameters (deadline, period, CPU reservation, and network bandwidth reservation) associated with the tasks to arrive at a configuration that meets the overall objectives. Nano-RK also provides APIs through which task parameters can be configured at run-time, but its use is discouraged, especially when a task represents hard real-time jobs. Figure 5 shows the Nano-RK architecture as given in [31].

### 7.2. Programming Model

One of the goals for Nano-RK was to facilitate application developers by allowing them to work in a familiar multitasking paradigm. This results in a short learning curve, rapid application development, and improved productivity. Since Nano-RK is a preemptive multitasking OS, it needs to save the context of the current task before scheduling the new task. Saving the state of each task results in large memory consumption and frequent context switches result in reduced performance and higher energy consumption.

In Nano-RK, each task has an associated Task Control Block (TCB). It is recommended that the TCB should be initialized during initialization and system image creation. The TCB stores the register contents, priority, period of recurrence, (CPU, network, sensors) reservation sizes, and port identifiers of the task. Based on the period of recurrence Nano-RK maintains two linked lists of TCB pointers to order the set of active and suspended tasks.

To provide real-time semantics, Nano-RK provides fully preemptive multitasking *i.e*., it ensures that the highest priority process in the ready queue always runs on the microcontroller.

We already discussed that each task has an associated TCB which contains the register and stack contents of the task, the task’s priority, the task’s CPU, network, and sensors reservations, the task’s port identifier, and its period. A single TCB requires significant memory, hence if there is large number of tasks in the system, the system may run out of memory space.

Each multithreaded OS needs to provide support for synchronization primitives so that correct state of shared data or other resources can be maintained. Nano-RK provides synchronization support in the form of mutexes and semaphores.

### 7.3. Scheduling

Nano-RK provides priority scheduling at two levels: priority scheduling at the process level and priority scheduling at the network level. In this section, we only discuss the scheduling algorithms that are being used in Nano-RK for process scheduling. To support real-time applications, Nano-RK uses a fully preemptive priority driven scheduling algorithm, *i.e*., at any given instance the highest priority task is scheduled by the operating system. A rate monotonic scheduling algorithm is used for real-time periodic tasks and the priority of the task is set statically based upon the period of the job: the shorter the period of the job, the higher is its priority. Since rate monotonic scheduling algorithm statically assigns priorities to tasks, Nano-RK recommends to configure task parameters offline.

Nano-RK also provides an implementation of rate harmonized scheduling for energy saving [32]. The main idea behind this scheduling algorithm is to eliminate CPU idle periods by grouping the execution of different tasks. Since initial arrival time, periodicity, and deadlines of tasks are known a priori, rate harmonized scheduling can be used to further save energy by eliminating idle cycles.

Nano-RK also provides an implementation of the priority ceiling algorithm to bind the blocking time encountered by a higher priority process due to priority inversion, *i.e*., the shared resource needed by the higher priority process is being used by a lower priority process. To circumvent priority inversion, each mutex is associated with a priority ceiling. Whenever a mutex is acquired by a task, the priority of the task is set equal to the priority ceiling of the mutex. Once, the mutex is released, the priority of the task is set equal to its original priority.

### 7.4. Memory Protection and Management

Nano-RK only provides support for static memory management, it does not support dynamic memory management. In Nano-RK, both the OS and applications reside in a single address space and to the best of authors’ knowledge Nano-RK does not provide any support to safeguard co-located OS and process address spaces.

### 7.5. Communication Protocol Support

Nano-RK provides a lightweight networking protocol stack that provides a communication abstraction similar to sockets. As in traditional network programming, an application that wants to send data can create a socket and then it can start communicate via that socket. Similarly, an application can bind and listen to a particular two bytes port number to receive data. To handle memory more efficiently, transmit and receive buffers are not managed by the OS, instead they are managed by the application. The rational presented for this is that it wastes memory if the OS reserves a considerable large space in memory for an application that only sends and receives a few bytes of data. Therefore, it is more appropriate to allow applications to manage their own send and receive buffers. The OS identifies application buffers by using the port number present in the packet header. The OS copies the received data into the application buffers using zero copy semantics. Once the data is placed into the application buffer, the application is notified accordingly. New incoming data is not copied into the application buffer until previously placed data is read by the application or the application explicitly allows the OS to do so.

A Time Synchronized Link Protocol for Energy Constraint Multi-hop Wireless Networks (RT-Link) [34] has been implemented in Nano-RK. The primary goal of RT-Link is to prolong a sensor network’s lifetime and to provide guarantees on end-to-end delay. RT-Link provides support for real-time applications through bounded end-to-end delay across multiple hops and collision free transmission. RT-Link is implemented over a TDMA link layer protocol, where each node’s transmission occurs in predefined time slots, allowing for energy savings. An RT-Link cycle consists of 32 frames and each frame consists of 32 time slots. The duration of each time slot is equal to 5 ms, enough to transmit a maximum size packet. The length of a single cycle is 5.12 s. There are two types of slots in RT-Link: Scheduled Slots (SS) and Contention Slots (CS). SS are allocated to those member nodes that require bounded end-to-end delay and the allocation of slots is carried out by the gateway *i.e*., the central controlling entity. Each member node sends its neighbor list to the gateway and the gateway assigns slots to the nodes depending on the network topology constructed through the neighbor list. A new node joins the network as a guest node and operates in contention slots. A node makes its reservation request to a gateway using CS. Once a SS is assigned, the node becomes a member node and operates during the SS period. A mobile node always operates in the CS period because its membership changes rapidly with time, disturbing the scheduling plan set by the gateway.

Since Nano-RK is a reservation-based OS, it provides APIs to applications so that they can reserve network bandwidth according to the application requirements. Nano-RK provides two sets of APIs: one for reserving sender side bandwidth and the other one for reserving receiver side bandwidth. In a given period, an application can only send or receive data according to its network bandwidth reservation status. This restriction can be relaxed in a soft real-time system if there is some slack bandwidth available in the system. In every new period the bandwidth reservations of each application is renewed. Nano-RK neither provides an implementation of multicasting routing algorithm nor does it provide an implementation of a group management protocol.

### 7.6. Resource Sharing

For shared resources such as memory, Nano-RK provides mutexes and semaphores for serialized access. To circumvent the priority inversion problem, Nano-RK provides an implementation of the Priority Ceiling algorithm. In addition, Nano-RK provides APIs to reserve system resources like CPU cycles, sensors, and network bandwidth.

### 7.7. Support for Real-time Applications

Nano-RK is a real-time operating system, hence it provides rich support for real-time applications. It supports real-time processes and its offline admission control procedure guarantees to meet deadline associated with each admitted real-time process. Nano-RK provides an implementation of real-time preemptive scheduling algorithms and tasks are scheduled using a rate monotonic scheduling algorithm. Moreover, Nano-RK provides bandwidth reservations for delay-sensitive flows and it claims to provide end-to-end delay guarantees in multi-hop wireless sensor network. Nano-RK is a suitable OS for use in multimedia sensor networks due to its extensive support provided to real-time applications.

### 7.8. Additional Features

In this section we discuss additional features provided by the Nano-RK OS.

- **Language Support**
  Nano-RK supports application development in the C language.
- **Supported Platforms**
  Nano-RK supports the following sensing platforms: MicaZ [16], and FireFly [33].
- **Documentation Support**
  Nano-RK documentation can be found on the Nano-RK home page at:http://www.nano-rk.org.

## 8. LiteOS

LiteOS [35] is a Unix-like operating system designed for WSNs at the University of Illinois at Urbana-Champaign. The motivations behind the design of a new OS for WSN are to provide a Unix-like OS for WSN, provide system programmers with a familiar programming paradigm (thread-based programming mode, although it provides support to register event handlers using callbacks), a hierarchical file system, support for object-oriented programming in the form of LiteC++, and a Unix-like shell. The footprint of LiteOS is small enough to run on MicaZ nodes having an 8 MHz CPU, 128 bytes of program flash, and 4 Kbytes of RAM. LiteOS is primarily composed of three components: LiteShell, LiteFS, and the Kernel. In the following subsections we discuss these three components and other features of LiteOS in detail.

### 8.1. Architecture

**Figure 6.**
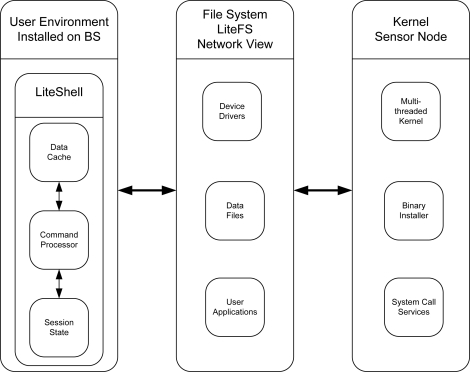
LiteOS Architecture.

LiteOS follows a modular architecture design. LiteOS is partitioned into three subsystems: LiteShell, LiteFS, and the Kernel. LiteShell is a Unix-like shell that provides support for shell commands for file management, process management, debugging, and devices. An interesting aspect of the LiteOS is its LiteShell that resides on a base station or a PC. This leverages to support more complex commands as the base station has abundant resources. The LiteShell can only be used when there is a user present on a base station. Some local processing is done on the user command (parsing a command) by the shell and then it is transmitted wirelessly to the intended sensor node. The sensor node does the required processing on the command and sends a response back, which is then displayed to the user. When a mote does not carry out the commands, an error code is returned. It is worth noting that Contiki and Mantis also provide a shell to interact with the motes but the implementation code resides on the mote, limiting the capabilities of the shell due to resource constraints. The second architectural component of LiteOS is its file system LiteFS. LiteFS mounts all neighboring sensor nodes as a file. LiteFS mounts a sensor network as a directory and then list all one hop sensor nodes as a file. User on the base station can use this directory structure just like the normal Unix directory structure and a user can also use legitimate commands on these directories. We discuss LiteFS in more detail later on. The third major component of LiteOS is the Kernel that resides on the sensor node. The LiteOS kernel provides concurrency in the form of multithreading, provides support for dynamic loading, uses round robin and priority scheduling, allows programmers to register event handlers through callback functions, and provides synchronization support. Figure 6 shows the architecture of LiteOS.

### 8.2. Programming Model

LiteOS is a multitasking OS and it supports multithreading. In LiteOS, processes run applications as separate threads. Each thread has its own memory space to avoid potential semantic errors that could occur during read and write in shared memory space. LiteOS also provides support for event handling. Application programmers can register event handlers using a callback facility provided by LiteOS.

To avoid potential race conditions, LiteOS provides atomic_start() and atomic_end() functions. Whenever shared data among different threads is accessed or modified, it is highly recommend to use these functions. The LiteOS documentation does not detail how this functions are internally implemented, *i.e*., whether they are disabling interrupts or using mutexes.

### 8.3. Scheduling

LiteOS provides an implementation of Round Robin scheduling and Priority-based scheduling. Whenever a task is added to the ready queue, the next task to be executed is chosen through priority-based scheduling. The tasks run to completion or until they request a resource that is not currently available. When a task requires a resource that is not available, the task enables interrupts and goes to sleep mode. Once the required resource becomes available, the appropriate interrupt is signaled and the task resumes it execution from where it had left. When a task completes its operation it leaves the kernel.

When there are no active tasks in the system, the sensor node goes to sleep mode. Before going to sleep mode the node enables its interrupts so that it can wake up at the proper event or time.

Since the LiteOS scheduler allows tasks to run until completion, there is a chance that a higher priority task enters the ready queue when a low priority task is completing its execution. In this scenario, a higher priority task may miss its deadline, therefore LiteOS is not an appropriate OS for real-time sensor networks.

### 8.4. Memory Protection and Management

Inside the kernel, LiteOS supports dynamic memory allocation through the use of C-like malloc and free functions. User applications can use these APIs to allocate and de-allocate memory at run-time. Dynamic memory grows in the opposite direction of the LiteOS stack. The dynamic memory is allocated from the unused area between the end of the kernel variables and the start of the user application memory blocks. This allows adjusting the size of dynamic memory as required by the application.

The LiteOS kernel compiles separately from the application, therefore the address space is not shared between the kernel and the application. Similarly, each user application has its separate address space. Processes and Kernel memory safety is enforced through separate address spaces.

### 8.5. Communication Protocol Support

LiteOS provides communication support in the form of files. LiteOS creates a file corresponding to each device on the sensor node. Similarly, it creates a file corresponding to the radio interface. Whenever there is some data that needs to be sent, the data is placed into the radio file and is afterward wirelessly transmitted. In the same manner, whenever some data arrives at the node it is placed in the radio file and is delivered to the corresponding application using the port number present in the data

At the network layer LiteOS supports geographical forwarding. Each node contains a table that can only hold 12 entries. This routing protocol is supported in LiteOS version 0.3. Unfortunately, detailed documentation on communication protocols supported by LiteOS is not available, hence there has been no indication about the protocols supported at the MAC, network and transport layer.

### 8.6. Resource Sharing

LiteOS suggest the use of APIs provided for synchronization whenever a thread wants to access resources that are shared by multiple threads. The LiteOS documentation does not provide any detail on how system resources are shared among multiple executing threads.

### 8.7. Support for Real-Time Applications

LiteOS does not provide any implementation of networking protocols that support real-time multimedia applications. It provides a priority-based process scheduling algorithm but once a process is scheduled it runs to completion. This can result in a missed deadline of a higher priority process that enters the ready queue once a low priority process has been scheduled.

### 8.8. Additional Features

In the section, we discuss additional features provided by the LiteOS.

- **Lite File System (LiteFS)**
  LiteOS provides support for a hierarchical file system called LiteFS. LiteFS provides support for both files and directories. LiteFS is partitioned in three modules. It keeps open file descriptors, memory allocation bit vectors, and information about flash memory layout in RAM. A second module resides in EEPROM and this module contains information about the hierarchical directory structure. Thirdly, it uses flash to store data. As in the Unix file system, files in LiteOS represent different entities, such as data, application binaries, and device drivers.
  The latest version of LiteFS supports eight file handlers in RAM and each handler consumes eight bytes. Therefore, at most eight files can be opened simultaneously. Two bit vectors are used to keep track of the current allocations in EEPROM and flash. The bit vector corresponding to the EEPROM consumes eight bytes and the bit vector corresponding to the data flash consumes 32 bytes. In total LiteFS requires 104 bytes inside the RAM.
  LiteFS mounts all single hop nodes to the file system just like mounting a USB device. It is important to note that since all single hop neighbors are mounted on a PC running some version of Linux OS, a user with access to the PC can copy or delete files on these nodes. The user can copy a new binary executable from the PC to a particular node and then it can issue an exec command to instruct the node to start executing the file.
- **Simulation Support**
  LiteOS supports wireless sensor networks simulations through AVRORA.
- **Language Support**
  LiteOS supports application development in the LiteC++ language.
- **Supported Platforms**
  LiteOS supports the following sensing platforms: MicaZ [16] and AVR series MCU [27].
- **Documentation Support**
  LiteOS documentation can be found on the LiteOS home page at: http://www.liteos.net.

## 9. Comparative Analysis

In this section, we summarize our survey of OSs for WSNs. Table 1 summarizes the previous discussions based on the common features: OS Architecture, Programming Model, Scheduling, Memory Management and Protection, Communication Protocol Support, Resource Sharing, and Support for Real-Time Applications.

From the above table it can be seen that few OSs provide support for real-time application. Some OSs provide support for priority scheduling while many others do not even provide support for this. Apart from Nano-RK, none of the surveyed OSs provides support for real-time applications at the communication protocol stack. It can also been seen that early OSs for WSNs emphasized on using an event driven programming paradigm. However, as programmers are more familiar with threading based programming paradigm, contemporary OSs for WSNs support the threading based programming model. Table 2, summarizes the miscellaneous features of surveyed OSs for WSNs.

## 10. Future Research Directions

Plenty of research has already been done on OSs for WSN, and it is still an active research domain. As it is a relatively new research area, and the range of possible WSN application domains and hence WSN applications is growing, there is still significant room for additional work. In addition, new hardware developments will lead to new classes of sensors, networking capabilities, and improved basic sensor platforms (memory, storage, CPU clock rates, *etc*.). The following are some issues that should be addressed to arrive at a mature, modern OS for wireless sensor nodes in future research.

### 10.1. Support for Real-Time Applications

There are many real-time application areas for WSN, e.g., in industry automation, chemical process monitoring, and multimedia data processing and transmission. Schedulers have been designed to support soft as well as hard real-time operations in some operating systems, but the effort is far from complete. In future, there is a need for scheduling algorithms that can accommodate both soft and hard real-time requirements of applications. Furthermore, with the emergence of WMSNs, a networking protocol stack is required that provides implementation of protocols that support some kind of differentiated services.

### 10.2. Secondary Storage Management

With the passage of time, new application areas for WSN are emerging and applications are requiring more and more memory. A typical databases application requires a secondary storage with sensor nodes. There are only few OSs that provide a file system to manage secondary storage. If we consider Moore’s Law, then in the not-to-distant future we can expect to have powerful sensor nodes with large secondary storage, therefore more research effort is required to build a scalable (distributed) file system for WSNs.

### 10.3. Virtual Memory Support

A sensor node has very limited RAM and applications are requiring more and more RAM. Therefore, in the future we need to introduce virtual memory concepts in OSs for WSNs. We need to devise virtual memory management techniques that are energy and memory-efficient.

### 10.4. Memory Management and Security

Little work has been done on memory management for WSN OSs. The primary reason behind this is that it has been assumed that only a single application runs on a WSN OS. In many contemporary OSs, like TinyOS and Contiki, the application program and kernel compiles together, thus sharing the same address space. This can be harmful, therefore an OS like LiteOS provides separate address spaces for different applications and the kernel. Such techniques need to mature further to support a multitude of (concurrent) applications on wireless sensor nodes.

### 10.5. Support for Multiple Applications

Contemporary OSs for WSNs have been developed with the assumption that only a single application will run on the mote. But with the emergence of new application areas of sensor networks this base assumption is being questioned. Let us consider the case of WMSN, in which sensor motes are equipped with a voice sensors (microphones), video sensors such as cameras, and scalar sensors. Hence on a single sensor node we may have an application that is using the video sensor, *i.e*., recording video, applying some image processing techniques on it and sending the compressed video to the base station. Similarly, we can have other applications that are making use of voices sensors and scalar sensors. Therefore, OSs need to accommodate multiple applications at the same time.

### 10.6. Robust Communication Protocol Stack

Many WSN OSs provide a communication API that allows applications to send and receive data. Underneath, these APIs use the communication protocol stack provided by the OS. There is no agreement on a unified protocol stack for sensor networks, hence almost each OS provides its own custom implementation of a communication protocol stack. As a consequence, no communication can occur between two motes using different OSs. In the recent past, Contiki has provided an implementation of a micro IPv6 stack for sensor networks. This enables sensors nodes to use IP addresses and communicate with other IP-enabled devices. Following the Contiki approach, TinyOS in its new version has provided support for IP that conforms to the 6LowPan standard. Providing an implementation of the micro IP protocol stack allows two motes using different OSs to communicate. However, this approach may lack the capabilities to adequately handle multimedia streams.

There is a need for research effort that designs and implemenst a suitable cross layer protocol for sensor networks. The protocol design needs to consider constraints on sensor nodes as well as new emerging application areas for sensor networks.

### 10.7. Security

The deployment of WSNs in battlefield requires stringent security features, therefore, OSs for WSN should provide mechanisms so that a user at base station can authenticate valid nodes in the network. Similarly, in case of queries and updates, nodes must be able to authenticate the originator.

### 10.8. Database Management System Implementation

The task of a sensor node is to sense, perform computations, transmit, and store data. In some sensor networks data is only send to the base station in response to a query, therefore a sensor node must be able to store data and understand the query language. A research effort is required to design a database management system for sensor nodes that suits well to their characteristics.

### 10.9. Localization and Clock Synchronization API Support

Considerable amount of work has been done in localizing the sensor node, as knowing the position of a sensor node at a given time has many applications. Similarly, many WSN applications (and some protocols) benefit from synchronized clocks. Therefore, an OS needs to provide support for these additional network protocols on the sensor nodes. For example, recent versions of TinyOS provide an implementation of FTSP, one of many clock synchronization protocols. As clock accuracy requirements are very application-specific, a better approach may be to allow developers to design their own protocol, with the OS providing appropriate APIs to facilitate in accomplishing this task.

### 10.10. APIs for Signal and Image Processing

As new application areas for WSNs in the field of image processing are emerging, a WSN OS needs to provide a rich set of basic image and signal processing APIs to facilitate the task of the application program developer.

## 11. Conclusions

In this paper, we have investigated the most widely used operating systems for WSNs. This paper helps to understand the characteristics of popular OSs for WSN in particular and embedded devices in general. Design strategies for various components of an OS for WSN have been explained and investigated, along with their relative pros and cons. Target application areas of different WSN OSs have been pointed out. We believe that the pros and cons of different design strategies presented here will motivate researchers to design more robust OSs for WSNs. Moreover, this survey will help the application and network designer to select an appropriate OS for their WSN applications.

## Figures and Tables

**Table 1.** Operating Systems Summary.

**OS/Feature**	**Architecture**	**Programming model**	**Scheduling**	**Memory Management and Protection**	**Communication Protocol Support**	**Resource Sharing**	**Support for Real-time Applications**
**TinyOS**	Monolithic	Primarily event Driven, support for TOS threads has been added	FIFO	Static Memory Management with memory protection	Active Message	Virtualization and Completion Events	No
**Contiki**	Modular	Protothreads and events	Events are fired as they occur. Interrupts execute w.r.t. priority	Dynamic memory management and linking. No process address space protection.	*u*IP and Rime	Serialized Access	No
**MANTIS**	Layered	Threads	Five priority classes and further priorities in each priority class.	Dynamic memory management supported but use is discouraged, no memory protection.	At Kernel Level COMM layer. Networking Layer isat user level. Application is free to use custom routing protocols.	Through Semaphores.	To some extent at process scheduling level (Implementatio n of priority scheduling within different processes types)
**Nano-RK**	Monolithic	Threads	Rate Monotonic and rate harmonized scheduling	Static Memory Management and No memory protection	Socket like abstraction for networking	Serialized access through mutexes and semaphores. Provide an implementation of Priority Ceiling Algorithm for priority inversion.	Yes
**LiteOS**	Modular	Threads and Events	Priority based Round Robin Scheduling	Dynamic memory management and it provides memory protection to processes.	File based communication	Through synchronization primitives	No

**Table 2.** Miscellaneous Features Summary.

**OS/Feature**	**Communication Security**	**File System Support**	**Simulation Support**	**Programming Language**	**Shell**
**TinyOS**	TinySec	Single level file system	TOSSIM	NesC	Not available
**Contiki**	ContikiSec	Coffee file system	Cooja	C	Unix-like shell runs on sensor mote
**MANTIS**	Not available	Not available	Through AVRORA	C	Unix-like shell runs on sensor mote
**Nano-RK**	Not available	Not available	Not available	C	Not available
**LiteOS**	Not available	LiteFS	Through AVRORA	LiteC++	Shell that runs on base station
